# The Predictive Value of the Boston Acute Stroke Imaging Scale (BASIS) in Acute Ischemic Stroke Patients among Chinese Population

**DOI:** 10.1371/journal.pone.0113967

**Published:** 2014-12-22

**Authors:** Yuanqi Zhao, Min Zhao, Xiaomin Li, Xiancong Ma, Qinghao Zheng, Xiaosheng Chen, Yinwing Lin, Yefeng Cai

**Affiliations:** 1 Department of Neurology, Guangdong Provincial Hospital of Chinese Medicine, Guangzhou, China; 2 National Clinical Research Center of Kidney Disease, State Key Laboratory of Organ Failure Research, Southern Medical University, Guangzhou, China; 3 Department of Neurology, JiangmenWuyi Traditional Chinese Medicine Hospital, Jiangmen, Guangdong, China; 4 Department of Neurology, Yue Bei people's Hospital, Shaoguan, Guangdong, China; 5 Department of Internal medicine, Meizhou Municipal Hospital of traditional Chinese Medicine, Meizhou, Guangdong, China; 6 Department of Neurology, Kang Mei Hospital, Jieyang, Guangdong, China; 7 Department of Chinese Medicine, Kwong Wah Hospital-The Chinese University of Hong Kong Chinese Medicine Clinical Research and Services Center, Hong Kong, China; Massachusetts General Hospital, United States of America

## Abstract

**Objective:**

Evaluate the predictive value of Boston Acute Stroke Imaging Scale (BASIS) in acute ischemic stroke in Chinese population.

**Methods:**

This was a retrospective study. 566 patients of acute ischemic stroke were classified as having a major stroke or minor stroke based on BASIS. We compared short-term outcome (death, occurrence of complications, admission to intensive care unit [ICU] or neurological intensive care unit [NICU]), long-term outcome (death, recurrence of stroke, myocardial infarction, modified Rankin scale) and economic index including in-hospital cost and length of hospitalization. Continuous variables were compared by using the Student t test or Kruskal-Wallis test. Categorical variables were tested with the Chisquare test. Cox regression analysis was applied to identify whether BASIS was the independent predictive variable of death.

**Results:**

During hospitalization, 9 patients (4.6%) died in major stroke group while no patients died in minor stroke group (p<0.001), 12 patients in the major stroke group and 5 patients in minor stroke group were admitted to ICU/NICU (p = 0.001). There were more complications (cerebral hernia, pneumonia, urinary tract infection) in major stroke group than minor stroke group (p<0.05). Meanwhile, the average cost of hospitalization in major stroke group was 3,100 US$ and 1,740 US$ in minor stroke group (p<0.001); the average length of stay in major and minor stroke group was 21.3 days and 17.3 days respectively (p<0.001). Results of the follow-up showed that 52 patients (26.7%) died in major stroke group while 56 patients (15.1%) died in minor stroke group (P<0.001). 62.2% of the patients in major stroke group and 80.4% of the patients in minor stroke group were able to live independently (P = 0.002). The survival analysis showed that patients with major stroke had 80% higher of risk of death than patients with minor stroke even after adjusting traditional atherosclerotic factors and NIHSS at baseline (HR = 1.8, 95% CI: 1.1–3.1).

**Conclusion:**

BASIS can predict in-hospital mortality, occurrence of complication, length of stay and hospitalization cost of the acute ischemic stroke patients and can also estimate the long term outcome (death and the dependency). BASIS could and should be used as a dichotomous stroke classification system in the daily practice.

## Background

Stroke has already been the leading cause of death and disability in Chinese adults [1], with a high occurrence and recurrence rate [2], [3]. The total cost of hospitalization for stroke patients was twice as much as per capita annual net income of China and still growing [3]. Many stratification tools and neurological scales were used to predict the prognosis of the patients which could help with formulating individualized treatment plans.

NIHSS (National Institute of Health stroke scale), mRS(modified Rankin Scale)and BI (Barthel Index) [4] were the most common and effective clinicometric and functional impairment scales of stroke patients while Alberta Stroke Program early computed tomography scale (ASPECT) [5] was the effective neuroimaging scale to evaluate the condition of stroke patients. However, there were some limitations. The severity of the disease was usually consistent with the score of NIHSS except some of the posterior circulation ischemia or accompanied with large vessel stenosis [6]–[9]. BI and mRS were more sensitive to assess the patients' post-stroke ability [10]–[14] while ASPECT scale was only scoring of parenchymal changes without information of arterial stenosis or occlusion.

BASIS is a neuroimaging-based ischemic stroke classification system which classifies the ischemic stroke into major or minor stroke, based on the volume, areas of the infarction and stenosis of the intracranial artery. It has been proved that BASIS could predict the outcome, hospitalization and expenses of stroke patients [15], [16]. Sillanpaa et al. compared several scales including BASIS, Clot Burden Score (CBS), cerebral blood volume(CBV)and ASPECTS and found that both BASIS and CBS had good predictive effect [17]. However, there was no information of long-term effect and no relative analysis of other influential factors such as age and severity of stroke, etc. STOPStroke study was a prospective cohort study comparing the predictive value of ASPECT, BASIS and NIHSS in the prognosis of ischemic stroke, the results showed that BASIS and NIHSS were independent predictive index of ischemic stroke while the combination was even better [18]. As we know, there was an obvious different distribution of intracranial artery stenosis in European countries and China [19], [20], so whether BASIS could be applied in Chinese stroke patients was still unknown. Our study is a retrospective study aiming to evaluate the predictive value of BASIS in acute ischemic stroke patients among Chinese population.

## Material and Methods

### Study Population

Subjects were acute ischemic stroke patients admitted to the neurological department of Guangdong Provincial Hospital of Chinese Medicine between August 2005 and July 2008. The inclusion criteria were (1) cerebral infarction verified by brain CT or MRI; (2) time from symptom onset was within 14 days; (3) MRA information obtained from the electronic medical system.

### Ethic Statement

Our study was approved by the ethic committee (2008 GL-37) of 2nd Affiliated Hospital of Guangzhou University of Chinese Medicine.Imaging Evaluation.

Patients were classified as having a major stroke based on BASIS which was when, a proximal cerebral artery occlusion was identified or when a large infarct was identified in major cerebral arteries by using either CT or MR angiography. Proximal cerebral artery occlusion was defined as an occlusion in the distal (intracranial) internal carotid artery, the proximal (M1 or M2) middle cerebral artery, or the basilar artery. If these arteries were not occluded, the presence of parenchymal abnormalities, such as a significant ischemic lesion in the middle cerebral artery territory or any lesion of the bilateral pons and/or bilateral thalami identified with noncontrast CT or diffusion MR imaging, patients were classified as having a major stroke. Patients not meeting these criteria were classified as having a minor stroke [21]. Whether patients had the artery stenosis or not was based on the CT or MR angiography (MRA). Two neuroimaging experts who were blinded to the patients' symptoms classified all patients into major or minor stroke by BASIS without knowing each other's conclusion. Once there was a disagreement, the third opinion was taken from another attending in the department and the decision was made based on the opinions of two out of three.

### Data extraction and Telephone follow up

Patients with diagnosis of cerebral infarction or ischemic stroke were pulled out from the medical record system. Then, the investigators verified the diagnosis according to CT/MRI and excluded the patients having stroke more than 14 days or without out MRA evaluation depending on the medical record. Finally, patients fulfilling the criteria were enrolled in our study.

Demographic information and medical history including hypertension, diabetes mellitus, hyperlipidemia, atrial fibrillation, smoking, prior stroke, prior transient ischemic attack and coronary artery disease was obtained. Patients' deaths, complications, admission to ICU/NICU during hospitalization as well as hospitalization cost and length of stay were retrospectively extracted from the electronic medical record. Clinical stroke severity was determined based on the NIHSS at admission.

Since we wanted to evaluate the predictive value of BASIS for the long-term outcome of the patients, 5 professionals of neurological department accepted the training of mRS evaluation and did the telephone follow-up to evaluate the living ability. And inquired whether the patients were alive or readmitted to hospital for myocardial infarction or recurrence of stroke at the same time.

### Outcome measurements

Short-term outcome included death, recurrence of stroke, occurrences of complications, being in ICU/NICU, during hospitalization as well as hospitalization cost and length of stay. Long-term outcome included death, recurrent stroke and living ability (mRS). Modified Rankin Scores were dichotomized to favorable (modified Rankin Score ≤2) or unfavorable (modified Rankin Score >2) outcomes [22], [23].

### Statistical analysis

Continuous variables were analyzed with a Student t test while categorical variables were analyzed using Chisquare test. P values <0.05 were considered statistically significant. Cox regression analyses were used to identify the hazard ratio of major stroke for total death. Model 1 adjusted age and gender, model 2 adjusted age, gender, history of hyperlipidemia, hypertension, diabetes, atrial fibrillation, prior stroke, prior transient ischemic attack, coronary artery disease, smoke and drink, model 3 adjusted the baseline NIHSS on the basis of model 2. All analyses were performed using Empower(R) (www.empowerstats.com, X&Y solutions, inc. Boston, MA) and R (http://www.R-project.org).

## Results

**Figure 1:**
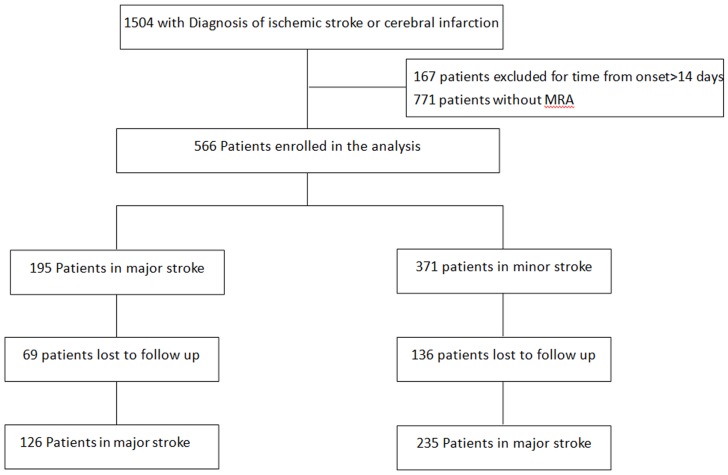
Flow Chart of the Patients Enrolled.

1504 patient were extracted from electronic medical system with the diagnosis of “ischemic stroke” or “cerebral infarction”. All patients had CT and/or MRI during hospitalization. The investigators verified the diagnosis by reviewing the neuroimaging materials. 167 patients had the stroke more than 14 days, 771 patients didn't had MRA evaluation during hospitalization. Finally, 566 patients were enrolled in the analysis. According to the evaluation of the neuroimaging experts, 195 patients were classified with major stroke and 371 patients with minor stroke (Fig. 1).

### Characteristic of the patients in Major stroke and Minor stroke group

The average age of patients in major and minor stroke group was 67.9±11.4 and 68.3±11.6 years old. 56.4% and 60.9% of patients in major and minor stroke group were male. Average NIHSS of patients with major and minor stroke was separately 5.2±3.9 and 3.4±2.8 (P<0.001). 65.1% of the patients in major stroke group and 76.8% of the patients in minor stroke group had NIHSS <8 while 34.9% of the patients in major stroke group and 23.2% in minor stroke group had NIHSS ≥9. 66 patients in major and minor stroke group and 81 patients in minor group had stroke history, 11 patient in major stroke and 4 patients in minor stroke group had history of auricular fibrillation (P = 0.001). (Table 1).

**Table 1:** Characteristic of patients in Major and Minor stroke group.

	Major	Minor	P value
N	195	371	
AGE	67.9±11.4	68.3±11.6	0.68
Gender(Male)	110 (56.4%)	226 (60.9%)	0.30
Hypertension	135 (69.2%)	242 (65.2%)	0.34
Diabetes	42 (21.5%)	71 (19.1%)	0.50
Coronary artery disease	21 (10.8%)	30 (8.1%)	0.29
Hyperlipidemia	9 (4.6%)	25 (6.7%)	0.31
Prior TIA	2 (1.0%)	8 (2.2%)	0.33
Prior Stroke	66 (33.8%)	81 (21.8%)	0.002
Atrial fibrillation	11 (5.6%)	4 (1.1%)	0.001
Drink	33 (16.9%)	77 (20.8%)	0.27
Smoke	66 (33.8%)	132 (35.6%)	0.68
NIHSS	5.2±3.9	3.4±2.8	<0.001

### Short-term outcome in Major stroke and Minor stroke group

During hospitalization, 9 patients(4.6%)died in major stroke group while no patients died in minor stroke group (P<0.001). Meanwhile, more patients in major stroke group were admitted in ICU (6.2% in major stroke group VS 1.3% in minor stroke group, P = 0.001). The occurrence rates of cerebral hernia, pneumonia and urinary infection were higher in major stroke group (P<0.05) (Table 2).

**Table 2:** Short–term outcome of Major stroke and Minor stroke group.

	Major	Minor	P
Death n%	9 (4.6%)	0 (0.0%)	<0.001
ICU use n%	12 (6.2%)	5 (1.3%)	0.001
Progressive stroke n%	15 (7.7%)	16 (4.3%)	0.09
Recurrent stroke n%	1 (0.5%)	1 (0.3%)	0.64
Epilepsy n%	1 (0.5%)	1 (0.3%)	0.64
Hernia n%	6 (3.1%)	0 (0.0%)	<0.001
Hemorrhagic transformation n%	2 (1.0%)	2 (0.5%)	0.51
Pneumonia n%	20 (10.3%)	18 (4.9%)	0.02
Urinary infection n%	20 (10.3%)	9 (2.4%)	<0.001
Bedsore n%	6 (3.1%)	6 (1.6%)	0.25
Gastric bleeding n%	1 (0.5%)	0 (0.0%)	0.17
Deep venous thrombosis n%	1(0.5%)	0 (0.0%)	0.167

### Economic index in Major stroke and Minor stroke group

The average length of stay in major stroke group was 21.3 days and 17.3 days in minor stroke group (P<0.001). The average duration in ICU was 5.6 days in major stroke group while 2.8 days in minor stroke group. Cost of hospitalization in major stroke group was 3100 US$ and 1740 US$ in minor stroke group (P<0.001). (Table 3).

**Table 3:** Economic index of Major stroke and Minor stroke group.

	Major	Minor	P
Cost (US$) ( 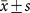 )	3100.6±4294.0	1740.7±1421.3	<0.001
Length of stay(days) ( 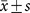 )	21.3±17.6	17.3±9.1	<0.001
ICU days	5.6±16.4	2.8±4.1	0.106
NICU days	8.09±1.744	4.35±5.142	0.589

### Long –term outcome in Major stroke and Minor stroke group

We evaluated mRS through telephone, 205 patients were lost because the loss of telephone number, change of telephone number and address, etc. Lost rate between major and minor stroke group was not statistically different (P = 0.648). Finally, 361 patients were included in the analysis of long-term outcome, among which 126 patients in major stroke group and 235 patients in minor stroke group.

Results of the follow-up showed that 43 patients died in major stroke group while 56 patients died in minor stroke group. 62.2% of the patients in major stroke group and 80.4% of the patients in minor stroke group had a favorable outcome (P = 0.002) (Table 4).

**Table 4:** Long-term outcome of Major stroke and Minor stroke group.

		Major	Minor	P
NO.		126	235	
Death n%		52 (26.7%)	56 (15.1%)	<0.001
mRS	0–2 n%	46 (62.2%)	144 (80.4%)	0.002
	3–5 n%	28 (37.8%)	35 (19.6%)	

There were 9 patients in major stroke group and 0 patients in minor stroke group died at discharge and another 43 patients in major stroke group and 56 patients in minor stroke group died during follow up. Together, there were 52 patients and 56 patients died in major and minor stroke group. The Cox regression showed that patients with major stroke had 80% higher of risk of death than patients with minor stroke even after adjusting of traditional atherosclerotic factors and NIHSS at baseline(HR = 1.8, 95%CI: 1.1–3.1) (Table 5).

**Table 5:** HRs (95% CI) of death according to subtype of BASIS.

Variable		BASIS		P value
		Minor	Major	
Death		52 (26.7%)	56 (15.1%)	
Cases(n)		195	371	
HR(95% CI)	Model A^a^	Ref.	1.9 (1.2, 3.0)	0.006
	Model B^b^	Ref.	1.9 (1.1, 3.2)	0.022
	Model C^c^	Ref.	1.8 (1.1, 3.1)	0.030

a Model A adjusted for age, sex;

b Model B adjusted for age, sex, history of dyslipidemia, hypertension, diabetes, auricular fibrillation, stroke, transient ischemic stroke, coronary heart disease, smoke and drink;

c Model C adjusted for age, sex, history of dyslipidemia, hypertension, diabetes, auricular fibrillation, stroke, transient ischemic stroke, coronary heart disease, smoke and drink and baseline NIHSS.

## Discussion

The results of our study demonstrated that BASIS was effective in predicting the outcome of patients with ischemic stroke, no matter for short-term or long-term outcome. Major stroke was the independent risk factors of death among patients with acute ischemic stroke. Meanwhile, BASIS could also predict the hospitalization cost and length of stay. It was thus worth to be applied in the clinical practice of the neurological department.

As we all know, there were so many factors related with the outcome of ischemic stroke, among which NIHSS, infarction volume, infarction location, large artery occlusion or stenosis were the most important ones. With the development of neuroimaging, more and more patients with ischemic stroke could be diagnosed as stroke with or without large artery stenosis. Professor Caplan suggested that large artery stenosis was the main predictor of prognosis and the effect of treatment [24]. Therefore, the perfect dichotomous tool of ischemic stroke should have the neuroimaging information, especially with the information of artery stenosis [25].

ASPECT score was a reproducible grading system developed to assess early ischemic changes (<3 hours from symptom onset) on pretreatment CT studies in patients with acute ischemic stroke of the anterior circulation [26]. This score was simple and reliable to identify stroke patients unlikely to make an independent recovery despite thrombolytic treatment. Clot burden score was a 10 points scale evaluating the ipsilateral intracraranial thrombus for the presence of contrast opacification in the complete cross-section of any part of M1, M2 or ICA. A score of 10 indicates absence of visible occlusion on CTA, a score of 0 indicates occlusion of all major intracranial anterior circulation arteries [27]. Both ASPECT and CBS were scales for anterior circulation arteries stroke while the former focus on the parenchyma and the later focus on the artery occlusion.

BASIS is a dichotomous neuroimaging-defined stroke severity classification system based on the neuroimaging. The rationale underlying the BASIS classification system is that large strokes are caused by major intracranial artery occlusion that persist for a sufficient period to cause infarction, that can be identified by CTA or MRA [21]. Unlike ASPECT and CBS, BASIS has information of both parenchyma and artery stenosis and can be used in stroke of anterior and posterior arteries area. Previous studies had proved BASIS could predict the outcome of stroke patients. Study of Mozqueda [21] et al enrolled 230 consecutive patients of acute ischemic stroke and compared the outcome of patients in major and minor stroke. The results showed that more patients survived and discharged to rehabilitation institutions with minor stroke and length of stay was much shorter in minor stroke group. After this study, Merino et al found some shortcomings including possible biases regarding the selection of imaging modality and timing of image acquisition, and the failure to take important clinical variables like age and stroke severity into account in the analysis which could influence the outcome of stroke [28]–[31]. This comment found that patients with CT were more likely to be diagnosed as major stroke than patients underwent MRI and patients with major stroke had a shorter time from symptom onset to imaging. Therefore, Merino suggested that BASIS must be validated in randomized trials before they can be used in routine clinical practice and in research studies [16]. STOPStroke program compared the effectiveness of BASIS, NIHSS and ASPECT on the outcome of ischemic stroke and found that both BASIS and NIHSS were independent predictors with similar sensitivity. Moreover, the predictive value is even higher if combining NIHSS (>10 and ≤10) and BASIS [9].

As we know, Asian population appeared to have higher prevalence of intracranial artery stenosis [32]. Whether these scales can be used among stroke patients in Chinese population was still unknown. This study was to evaluate the predictive value of Boston Acute Stroke Imaging Scale BASIS in acute ischemic stroke in Chinese population.

The results of our study suggested that there were more in-hospital deaths and complications in major stroke group than minor stroke group, while hospitalization cost and length of stay were also higher in major stroke group. In addition, the follow-up showed that long-term mortality rate was also higher in major stroke group while the independent rate in minor stroke group was higher. The cox regression showed that patients with major stroke had higher risk of death than patients with minor stroke (HR = 1.8 (95% CI: 1.1, 3.1) after adjusting traditional risk factors of atherosclerosis and NIHSS at baseline. It implied the BASIS was the independent predictors of patients with acute ischemic stroke in Chinese population.

Of course there are some limitations in the study. The main limitation is ours study was a retrospective design from a single center. Approximately 36% of patients were lost in the follow up which may have impacted our model if they had been included. Another limitation was about the evaluation of artery stenosis. Although all of the patients had brain CT/MRI, only 42% of the patients had MRA.

This study was investigated retrospectively which needs further prospective validation. We do feel BASIS will be a useful tool for the prediction of death, independent ability, hospitalization cost and length of stay which will help with the clinical decision in patients with ischemic stroke.

## Conclusion

BASIS can predict in-hospital mortality, occurrence of complication, length of stay and hospitalization cost of the ischemic stroke patients and can also estimate the long term outcome (death and the dependency). BASIS could and should be used as a dichotomous stroke classification system in the daily practice.
