# Correction: The Predictive Value of the Boston Acute Stroke Imaging Scale (BASIS) in Acute Ischemic Stroke Patients among Chinese Population

**DOI:** 10.1371/journal.pone.0126045

**Published:** 2015-05-12

**Authors:** Yuanqi Zhao, Min Zhao, Xiaomin Li, Xiancong Ma, Qinghao Zheng, Xiaosheng Chen, Yinwing Lin, Yefeng Cai

The first two authors, Yuanqi Zhao and Min Zhao, should not have been attributed equal contribution to this work. Min Zhao should be listed as a corresponding author. Dr. Min Zhao’s email address is: cassiesandra@163.com.

There is an error in Fig 1. On the bottom-right, “235 Patients in major stroke” should be “235 Patients in minor stroke.” Please see the corrected Fig 1 here.

**Fig 1:**
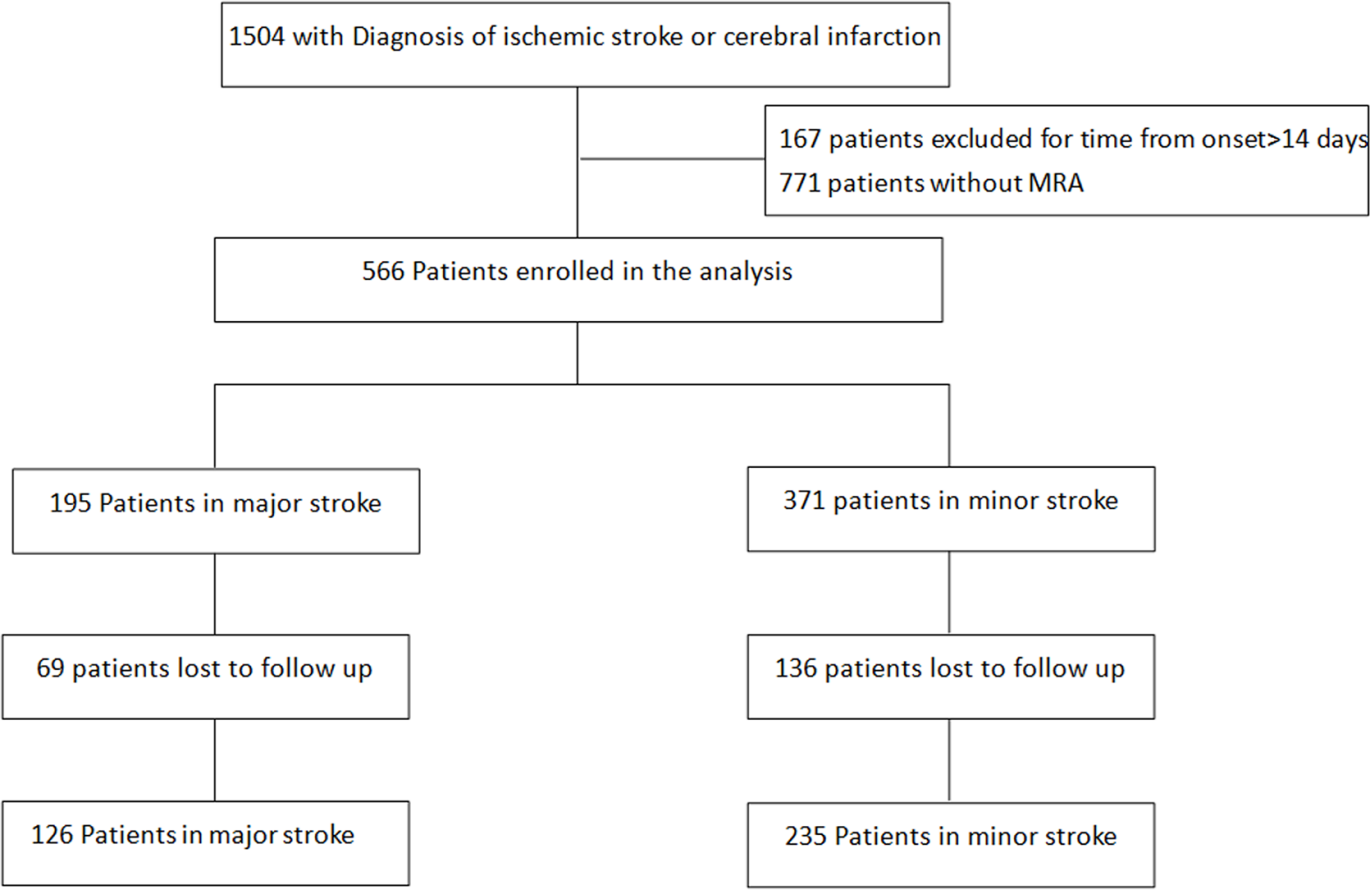
Flow Chart of the Patients Enrolled.
